# Breast cancer in togolese women: immunohistochemistry subtypes

**DOI:** 10.1186/s12905-020-01130-2

**Published:** 2020-11-23

**Authors:** Ablavi Adani-Ifè, Koffi Amégbor, Kwamé Doh, Tchin Darré

**Affiliations:** 1 Department of Oncology, Sylvanus Olympio University Teaching Hospital, BP 57, Lomé, Togo; 2 Department of Pathology, University Teaching Hospital of Lomé, BP 57, Lomé, Togo

**Keywords:** Breast cancer, Molecular subtype, Advanced stage, Immunohistochemistry, Togo

## Abstract

**Background:**

Molecular classification of breast cancer is an important factor for prognostic and clinical outcomes. There are no data regarding molecular breast cancer subtypes among Togolese women. The objective of this study was to evaluate the expression of ER, PR, HER2, and molecular subtypes of breast cancer receptors in Togolese patients and to establish the correlation between clinical and histological data and molecular types.

**Methods:**

Clinicopathologic data of patients were collected from clinical records. Immunohistochemistry biomarkers (ER, PR, and HER2) were assessed in patients who have been diagnosed with invasive breast cancer from March 2016 to March 2020 in the department of oncology. The analysis of variance and the Chi-square Test was used to analyze the data.

**Results:**

A total of 117 cases were collected. The mean age of patients was 52.05 ± 12.38 with an age range of 30 to 85 years. Half of the patients were over 50 years old and the majority (70.9%) was postmenopausal. More than half of patients (52.1%) presented with T3-T4tumors.The most common histologic subtype of breast cancer was invasive ductal carcinoma of no special type (95.7%). Tumors grade 2 were predominant (51.3%) followed by grade 3 (42.7%). Advanced carcinomas were found in 69 patients (59%). The percentage of ER+, PR+, and HER2 positive tumors was 54.7%, 41%, and 15.4% respectively. The predominant molecular subtype was Triple negative (37.6%), followed by Luminal A (30.8.7%), Luminal B subtype (23.9%), and HER2 enriched (7.7%). There was a significant association between stage and breast cancer subtypes (p 0.025), histologic grade, and subtype (p < 0.0001) but no correlation was found with age, menopausal status, and tumor size.

**Conclusion:**

Breast carcinoma in our patients are high grade tumors and are diagnosed at an advanced stage. Triple negative and Luminal A are the two predominant breast cancer subtypes in Togolese women. Consequently, Receptor testing availability should be a priority to offer the best breast cancer treatment.

## Background

Breast cancer is the most common malignancy in women and the leading cause of cancer mortality worldwide [1]. Breast cancer is a heterogeneous disease displaying clinical, pathological, and molecular varieties with various prognostic and therapeutic implications [2, 3].

Perou and colleagues [2] clustered breast cancer based on DNA microarray signature into Luminal A, Luminal B, HER2 enriched, Basal-like, and normal-like. Following this investigation, many studies classified Breast cancer molecular subtype using Immunohistochemistry (IHC) surrogate markers in a similar way to DNA microarray clustering [4–6]. With IHC, Breast cancer is classified into four groups based on the IHC profile of Estrogen Receptor (ER), Progesterone Receptor (PR) and Human Epidermal growth factor Receptor2 (HER2) expression, positive ( +) and/or negative (−) [5, 7, 8].

Breast cancer receptor status has major implications for breast cancer prevention strategies and patient management in clinical settings but the rate of these receptors in breast cancer varies from region to region.

In Togo, Breast cancer is the first cancer in women and is currently a major public health problem [9, 10]. However, there is no published data on molecular breast cancer subtypes in Togolese women. This study aimed to evaluate the expression of ER, PR, HER2, and receptor molecular subtypes of breast cancer in Togolese and to analyze the correlation between clinical and histologic markers and molecular subtypes.

## Patients and methods

### Patients

This is a retrospective and descriptive study of patients with a confirmed diagnosis of breast cancer in the oncology department of the Sylvanus Olympio Teaching Hospital of Lomé, from March 2016 to March 2020 (4 years). Togo is a small country of 56,600Km2, with an estimated population of 7,200,000, located between Ghana in the west and Benin in the east.

Data were extracted from patient records. Breast cancer has been classified according to the World Health Organization 2012 (WHO 2012). Histological classification was performed using the Nottingham classification system and staging according to the 8th edition of the AJCC classification of 2017.

### Immunohistochemistry tests

The study material consisted of biopsies and operating pieces fixed in 10% buffered formalin and came from various health structures in the country.

Immunostaining was done for Estrogen Receptor (ER) Progesterone Receptor (PR), Human Epidermal growth factor Receptor2 (HER2) and Ki-67 count using a Ventana Benchmark immunostainer using the manufacturers supplied antibodies. ER and PR positive nuclei greater than 1% were considered hormone receptor positive. HER2 was scored based on a 0 to 3 scale according to the criteria set by ASCO (American Society of Clinical Oncology) [11]. In the final analysis, a score of 3+ was considered overexpressed or positive and a score ≤ 2 as negative. Fluorescence in situ hybridization for HER2 amplification was not performed.

Breast cancer subtypes were defined according to the IHC expression of ER/PR/HER2 and Ki-67 count as follow:

$${\text{Luminal A }}\left( {{\text{ER}} + /{\text{PR}} + ,{\text{ HER2}} - ,{\text{ Ki}} - {67} \le { 14}\% {\text{ or grade 1 or 2 tumor grading}}} \right),$$

$${\text{Luminal B }}\left( {{\text{ER}} + /{\text{PR}} + ,{\text{ HER2}} + {\text{ or ER}} + /{\text{PR}} + ,{\text{ HER2}} - ,{\text{ Ki}} - {67} > { 14}\% {\text{ or grade3}}} \right),$$

$${\text{HER2 enriched }}\left( {{\text{ER}} - ,{\text{ PR}} - ,{\text{ HER2}} + } \right),$$

$${\text{Triple negative }}\left( {{\text{ER}} - ,{\text{ PR}} - ,{\text{ HER2}} - } \right).$$

### Statistic

Statistical analysis and data processing was performed with the software SPSS version 20.

Chi-square tests and the analysis of variance (ANOVA) were used to determine the correlations. P-value < 0.05 was considered statistically significant. The data were reported as frequencies for menopausal status, histological type, tumors grade, ER/PR status, and HER2 expression and as means for patient's age at presentation.

## Results

### Epidemiological and clinical data

From Mars 2016 to Mars 2020, 312 new breast cancer patients were registered in the department of oncology. Immunohistochemistry studies were performed on 117 patients who were included in this study.

The mean age of patients was 52.05 ± 12.38 with an age range of 30 to 85 years; 8 patients (6.8%) were less than 35 years old and 64 patients (54.7%) were more than 50 years old.

Eighty-three patients (70.9%) were postmenopausal at presentation. The tumor involved the right breast in sixty patients (51.3%) and the left in 55 patients (47%), two patients (1.7%) had bilateral breast cancer. More than half of the patients had tumor size greater than 5 cm (T3 and T4 tumors 52.1%).

### Pathological data

The predominant histologic subtype of breast cancer was invasive ductal carcinoma of no special type (NST) (95.7%). Invasive lobular carcinoma accounts for less than 3%.

According to the Nottingham grade classification, tumors were classified as grade 1 in 7 patients (6%), as grade 2 in 60 patients (51.3%), and as grade 3 in 50 patients (42.7%). The histological status of lymph nodes was determined for 78 patients (66.7%) among which 49 patients (41.9%) had positive lymph nodes.

Advanced carcinomas were found in 69 patients (59%) as the tumor clinical stage on the first diagnosis showed that 42 patients (35.9%) are at stage III and 27 patients (23.1%) are at stage IV. Patients characteristics are summarized in Table 1.

**Table 1:** Clinicopathologic characteristics of patients

Variables	Numbers of patients (%)
*Age*	
Mean age ± SD	52.05 ± 12.38
≤ 35 years	8 (6.8)
≥ 50 years	64 (54.7)
*Menopausal status*	
Premenopausal	34 (29.1)
Postmenopausal	83 (70.9)
*Histologic type*	
IDC NOS	112 (95.7)
Lobular	3 (2.6)
Medullary	1(0.9)
Mucinuous	1(0.9)
*Nottingham grade*	
1	7 (6)
2	60 (51.3)
3	50 (42.7)
*Tumor size*	
T1	10 (8.5)
T2	46 (39.3)
T3	26 (22.2)
T4	35 (29.9)
*Lymph nodes status*	
N0	29 (24.8)
N1	27 (23.1)
N2	10 (8.5)
N3	12 (10.3)
Nx (undetermined)	39 (33.3)
*Stage*	
I	3 (2.5)
II	45 (38.5)
III	42 (35.9)
IV	27 (23.1)

*IDC NOS* invasive ductal carcinoma of no special type

For immunohistochemistry, 36.8% of receptor testing was conducted on biopsy material and the remainder on mastectomy tissue.

Overall, the immunohistochemical study showed that invasive breast cancer cases were ER positive in 64 patients (54.7%), PR positive in 48 patients (41%), and HER2 positive in 18 patients (15.4%). Forty-eight patients (41%) were positive for both estrogen and progesterone receptors expressions. Half of the HER2 positive cases (50%) were ER+ and PR+ .

Therefore 36 tumors (30.8%) were classified as Luminal A, 28 (23.9%) as Luminal B, 9 (7.7%) as HER2 overexpressing, and 44 (37.6%) as Triple Negative Breast Cancer (TNBC). (Table 2).

**Table 2:** Expression of ER, PR and HER2 in cases and distribution of breast cancer molecular subtypes

Variables	Number of patients (%)
*Estrogen receptor status*	
Positive	64 (54.7)
Negative	53 (45.3)
*Progesterone receptor status*	
Positive	48 (41)
Negative	69 (59)
*HER2 Status*	
Positive	18 (15.4)
Negative	99 (84.6)
*Molecular subtype*	
Luminal A	36 (30.8)
Luminal B	28 (23.9)
Triple Negative	44 (37.6)
HER2 enriched	9 (7.7)

### Relationship between molecular subtype and clinicopathologic factors

The Mean age at diagnosis was the youngest for HER2 enriched tumors (46.3 years). Twenty patients (45.4%) with TNBC were below fifty years old whereas more than half of patients in the Luminal A group were over 50 years old. Age at diagnosis distribution by subtype is shown in Table 3. Most patients with Luminal A (80.5%) were postmenopausal.

**Table 3:** Correlation of clinicopathologic parameters with breast cancer subtypes

Variables	Luminal A N = 36 n (%)	Luminal B N = 28 n (%)	Triple negative N = 44 n (%)	HER enriched N = 9 n (%)	P value
Mean age (years)	54.14 ± 12.7	51.07 ± 13.19	52.16 ± 12.44	46.33 ± 6.30	0.377
*Age group (years)*					
< 40	1 (2.8)	5 (17.9)	8 (18.1)	1 (11.1)	0.172
[40–50]	14 (38.9)	6 (21.4)	12 (27.3)	6 (66.7)	
[50–60]	12 (33.3)	10 (35.7)	12 (27.3)	2 (22.2)	
≥ 60	9 (25)	7 (25)	12 (27.3)	0	
*Menopausal status*					
Premenopausal	7 (19.4)	10 (35.7)	13 (29.5)	5 (44.4)	0.163
Postmenopausal	29 (80.6)	18 (64.3)	31 (70.5)	4 (55.6)	
*Stage*					
I	3 (8.3)	0	0	0	0.025
II	15 (41.7)	12 (42.9)	18 (40.9)	0	
III	15 (41.7)	7 (25)	15 (34.1)	5 (55.6)	
IV	3 (8.3)	9 (32.1)	11 (25)	4 (44.4)	
*Grade*					
1	4 (11.1)	0	3 (6.8)	0	< 0.0001
2	29 (80.6)	7 (25)	17 (38.6)	7 (77.8)	
3	3 (8.3)	21 (75)	24 (54.6)	2 (22.2)	
*Tumor size*					
T1	6 (16.7)	1 (3.6)	3 (6.8)	0	0.078
T2	16 (44.4)	13 (46.4)	17 (38.7)	0	
T3	9 (25)	7 (25)	10 (22.7)	0	
T4	5 (13.9)	7 (25)	14 (31.8)	9 (100)	

The majority of TNBC were grade 3 and had larger tumor sizes while patients with Luminal A had well to moderately differentiated tumors and smaller tumor sizes. HER2 subtype had the highest percentage of T4 tumors (100%).

The Luminal A breast cancer patients were more diagnosed at an early stage compared to those of triple negative and HER2 subtypes which presented a high proportion of advanced tumors.

There was a significant association between stage and breast cancer subtypes (p 0.025), histologic grade, and subtypes (p < 0.0001) but no correlation was found with age, menopausal status, and tumor size (Table3).

## Discussion

The effective management of patients with breast cancer needs knowledge of the hormone receptor status and the HER2 overexpression.

This study is the first conducted in Togo, which determines the molecular groups of breast cancer based on the IHC expression of ER, PR, and HER2.

In this retrospective study, IHC was performed in only the third (37.5%) of breast cancers diagnosed from 2016 to 2020 because of the unavailability and the high cost of receptor testing. As in our country, access to IHC remains limited in most countries of Sub-Saharan Africa [12–15].

The mean age at diagnosis was 52.05 ± 12.38 years with an age range between 30 and 85 years, with half of the patients having more than 50 years old.

This age at presentation in our current study is in agreement with a previous Togolese study where the mean age at diagnosis of cancer was 50 years [10]. This is also similar to the mean age reported in Ghanaian patients at Korle bu Hospital [16] and in Indian women [17] but different from the relatively younger age of women with breast cancer reported in several studies in others countries in Africa [12, 18–22].

In this study, the majority of patients (70.9%) were postmenopausal at presentation, this is contrary to findings from other African studies where the majority of women were premenopausal [14, 22–26].

As observed in most breast cancer studies worldwide, invasive ductal carcinoma was the most dominant histological type of tumor in Togolese patients.

Our study revealed that less than 10% of women with breast cancer have well-differentiated tumors, 42% had positive lymph nodes and the majority had T3 or T4 tumors stage. These unfavorable clinicopathological characteristics such as high grade, large tumors size, axillary lymph node involvement, and advanced stage are the same as those reported in several studies [14, 15, 17, 21, 26, 27]. The advanced stage of diagnosis in our patients could be explained by the delay of consultation, the absence of a national breast cancer screening program in the population, the poor health care facilities, and the use of traditional medicine.

A recent systematic review and meta-analysis by Eng [28] reported most breast cancer cases in Africa as being Hormone Receptor positive. Nevertheless, the positivity of hormonal receptors in breast cancer remains varied and heterogeneous in countries.

In the present study, positive ER immunostaining was found in 54.7% of cases. Our result is similar to that reported by Effi in Ivory Coast [23]. However, our finding is lower than those reported in western countries [5] and Asia [29, 30]. The proportion of patients expressing ER (54.7%) is superior to those of PR positive (41%). The same observation was reported by different authors [12, 23, 29, 31].

Our findings in the current study, have implications for the management of breast cancer in Togo.

Indeed, in the past, patients with breast cancer were treated blindly with tamoxifen. However, forty-five percent (45%) of our patients are ER/PR negative and may not be suitable for hormonal therapy. Also, the high frequency of postmenopausal women in this study points out that hormonal therapy should be given according to the menopausal status for adequate care.

HER2 overexpression was seen in 15.4% of patients. This result is in agreement with the literature data which reported 15–20% of HER2 positive in invasive breast cancer [32]. Our result is similar to the finding reported in Tanzania [15] and Ivory Coast [23] but is different from the rate found in Ghanaian women [16, 24].

Even if our result was included in the interval rate of HER2, it could have been underestimated as FISH was not performed to ascertain the true HER2 status of tumors with an equivocal IHC score of 2+.

In our study, the distribution of molecular subtypes found a predominance of Triple Negative Breast Cancer (37.6%) followed by Luminal A subtype (30.8%), luminal B (23.9%), and HER2 enriched (7.7%).

This distribution is different from those reported in north African countries [33–35] were Luminal A was the most common subtype.

In our patients, the proportion of TNBC was 37.6%. Our result is lower compared with that noted for Nigerian (47.6%) [36] and Senegalese women (46.7%) [22] but much higher as compared with those reported from Ivory Coast (32.1%) [23], Angola (31.4%) [20], and Ethiopia (23%) [25]. However, our results may be influenced by technical issues, particularly the duration and the quality of fixation.

Many studies have reported that TNBC is the dominant phenotype in native African women [37–40] and African Americans [5, 41, 42] compared with white women. The TNBC is considered more common in younger women and is associated with aggressive clinicopathologic characteristics [27, 38]. Our findings corroborate these reports.

Luminal A subtype which is a less aggressive type of breast cancer was found in 30.8% of our patients. This rate was lesser than that reported in Saudi Arabia [29], India [17], and Western countries [5]. As expected, the Luminal A subtype was associated with favorable clinic and biological characteristics.

Twenty-eight tumors (23.9%) were classified as Luminal B subtype among which the third were Luminal B HER2 positive.

Our result is lower compared with the finding reported by Hadgu in Ethiopian women where Luminal B was the second most prevalent subtype (26%) [25] but much higher as compared with those reported from Senegal (4.6%) [22] and Angola (7.9%) [20].

HER2 enriched tumors are known to be associated with particular poor breast cancer outcomes but the use of HER 2 targeted therapy improves survival among breast cancer patients whose tumors overexpress HER2.

HER2 enriched subtype was found in 7.7% of our patients. A similar rate has been reported by Effi in Ivorian women [23]. In comparison with the other subtypes, HER2 enriched tumors were observed to be associated with larger tumor size.

The breast cancer subtypes were correlated with the stage (p = 0.025) and the histological grade (p < 0.0001). A significant association between histological grade with breast cancer has also been reported in Moroccan [19], in Sudanese and Eritrean women [43].

In several studies, some authors reported a significant association between molecular subtype and age [25, 33], tumor size [43], and menopausal status [33] but in this current study, we did not find any correlation between molecular subtype and age, tumor size or menopausal status.

This study demonstrated interesting observations about breast cancer in Togo. However, some limitations should be mentioned such as the unavailability of the cellular marker Ki 67 for all patients, the lack of evaluation for HER2 equivocal results using Fluorescence in situ, and the absence of cytokeratin5/6 to identify the different subset of triple negative cancer.

## Conclusion

For the first time, we report the distribution of molecular breast cancer subtypes and their associations with some clinicopathological characteristics in Togolese women. Breast carcinoma in our patients are high grade tumors and are diagnosed at an advanced stage. Hormone receptors were positive in more than half of the patients. The two predominant molecular subtypes are Triple negative and Luminal A. The histological grade and tumor stage are significantly associated with tumor subtypes. This study emphasizes the need for introducing the receptor testing in our routine clinical practice to offer the best breast cancer treatment.

## Data Availability

The datasets used and/or analyzed during the current study are available from the corresponding author on reasonable request.

## Abbreviations

ER: Estrogen receptor
PR: Progesterone receptor
HER2: Human epidermal growth factor receptor2
IHC: Immunohistochemistry
ANOVA: The analysis of variance
IDC NOS: Invasive ductal carcinoma of no special type
TNBC: Triple negative breast cancer
